# Monkeypox virus and type 1 diabetes: a molecular insight into inflammatory signaling and β-cell autoimmunity

**DOI:** 10.1186/s12985-025-02822-7

**Published:** 2025-06-14

**Authors:** Yahya A. Almutawif, Haydar M. Al-kuraishy, Ali I. Al-Gareeb, Ali K. Albuhadily, Hamza M. A. Eid, Athanasios Alexiou, Marios Papadakis, Mohammed E. Abo‑El Fetoh, Gaber El-Saber Batiha

**Affiliations:** 1 Department of Clinical Laboratory Sciences, College of Applied Medical Sciences, Taibah University, Madinah, 42353 Saudi Arabia; 2 Department of Clinical Pharmacology and Medicine, College of Medicine, Mustansiriyah University, Baghdad, Iraq; 3 Jabir ibn Hayyan Medical University, Al-Ameer Qu./ Najaf, Po. Box (13), Kufa, Iraq; 4 Department of Research & Development, Funogen, Athens, 11741 Attiki Greece; 5 University Centre for Research & Development, Chandigarh University, Chandigarh-Ludhiana Highway, Mohali, Punjab India; 6 University Hospital Witten-Herdecke, University of Witten-Herdecke, Heusnerstrasse 40, 42283 Wuppertal, Germany; 7 Department of Pharmacology and Toxicology, Faculty of Pharmacy, Egyptian Russian University, 11829 Badr City, Cairo Egypt; 8 Department of Pharmacology and Therapeutics, Faculty of Veterinary Medicine, Damanhour University, Damanhour, AlBeheira, 22511 Egypt

**Keywords:** Monkeypox, Type 1 diabetes, Autoimmunity, Immune dysregulation, Viral infections, Pancreatic β-cells, Inflammation, Cytokines, COVID-19, Diabetogenic viruses

## Abstract

The current worldwide pandemic of monkeypox (MPXV) elicited apprehensions over its possible long-term health ramifications. Recent data indicate a potential association between MPXV infection and the onset of autoimmune disorders, such as type 1 diabetes (T1D). This article analyzes the possible methods for MPXV infection to induce autoimmune responses and damage pancreatic β-cells. We examine from published articles the possible MPXV-induced immune dysregulation and inflammation in the development of T1D. Utilizing insights from other diabetogenic viruses, such as enterovirus and SARS-CoV-2. Currently, the association between MPXV and the development of T1D is scarce. Therefore, additional studies are essential for establishing a conclusive causal link between MPXV and T1D.

**Graphical abstract:**

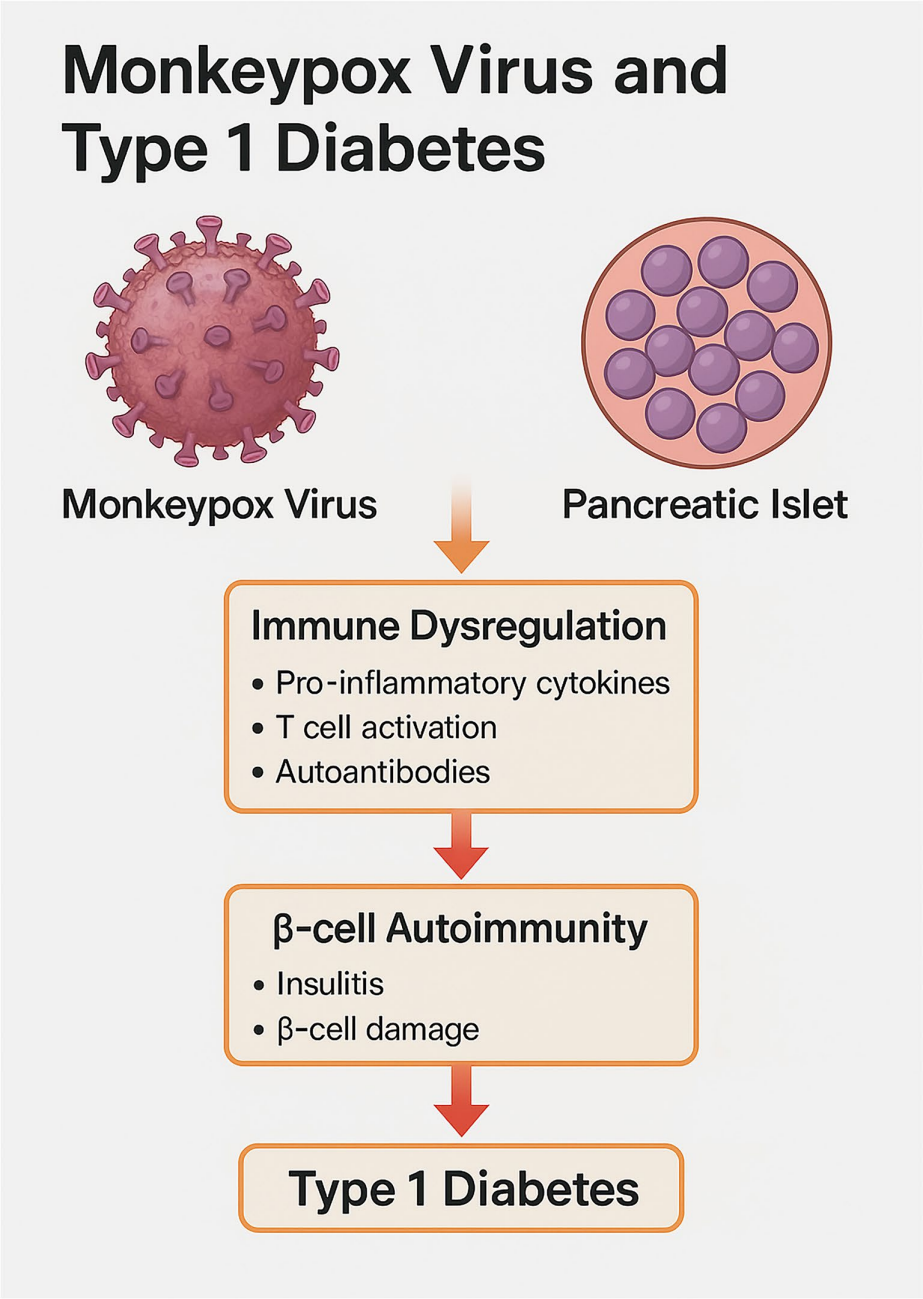

## Introduction

**Fig. 1:**
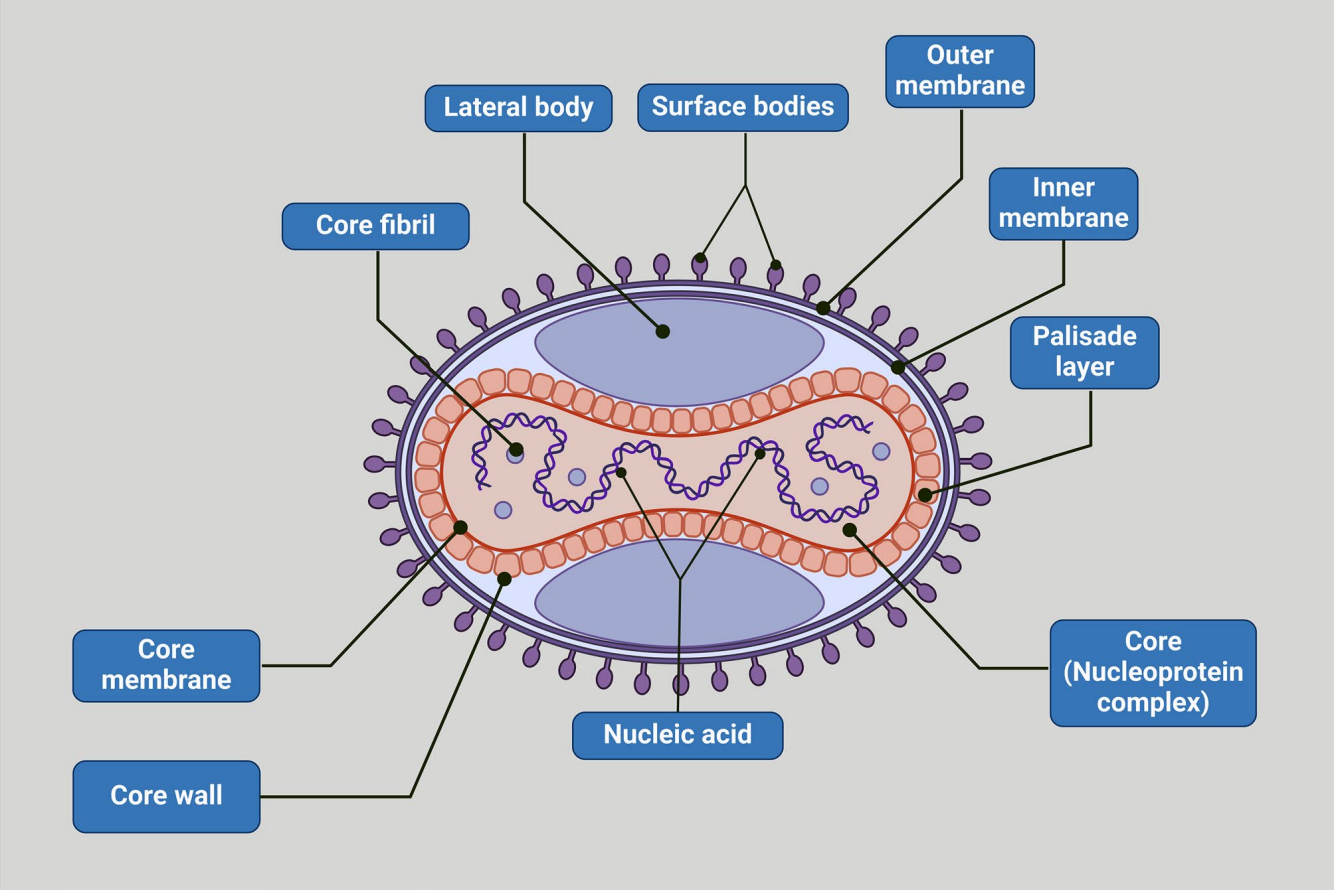
Viral structure of MPXV

Monkeypox virus (MPXV) was discovered in 1958 by virologist Preben C. A Magnus while studying smallpox-like diseases in laboratory monkeys [1]. According to the tracking of the origin of MPXV, it was suggested that this virus was dated since 1899 [2]. In addition, many reports highlighted that MPXV was found in Egyptian mummies [3, 4]. The first reported case of MPXV was in the Democratic Republic of Congo (DRC) in 1970 as a suspected smallpox case [5]. The MPXV is a double-strand DNA virus belonging to the *Orthopoxvirus* genus and *Poxviridae* family [Figure 1]. The MPXV is identical to the old Word *Orthopoxvirus*, which includes smallpox, vaccinia virus, and cowpox. Smallpox and MPXV are regarded as potential bioweapons [6].

The MPXV is a zoonotic disease, endemic in Africa, that can infect humans through direct contact with infected animals such as monkeys, rodents, prairie dogs, hedgehogs, squirrels, anteaters, and shrews. The virus then enters host cells, evading immune detection [7]. Therefore, MPXV is misnamed as the monkeys are not the main reservoir of MPXV [8]. Thus, renaming it as animal pox is better than human smallpox, which only infects humans. However, the MPXV name has been considered since the discovery of this disease in monkeys. In addition, human-to-human transmission of MPXV is mediated by direct close contact with infected patients [9]. Airborne transmission of this virus is involved in the secondary transmission of MPXV, which becomes a more transmissible virus [10]. In addition, sexual transmission of MPXV is considered a possible method of transmitting the virus from one person to another [11]. The human MPXV incubation phase lasts for 10–14 days. Afterwards, the infectious period extends as skin rash and lymphadenopathy develop [12]. The risk factors for MPXV are non-vaccination against smallpox, contact with animals, women, health providers, contact with infected subjects, ingestion of uncooked meat of infected animals, and immune-compromised subjects [13].

**Fig. 2:**
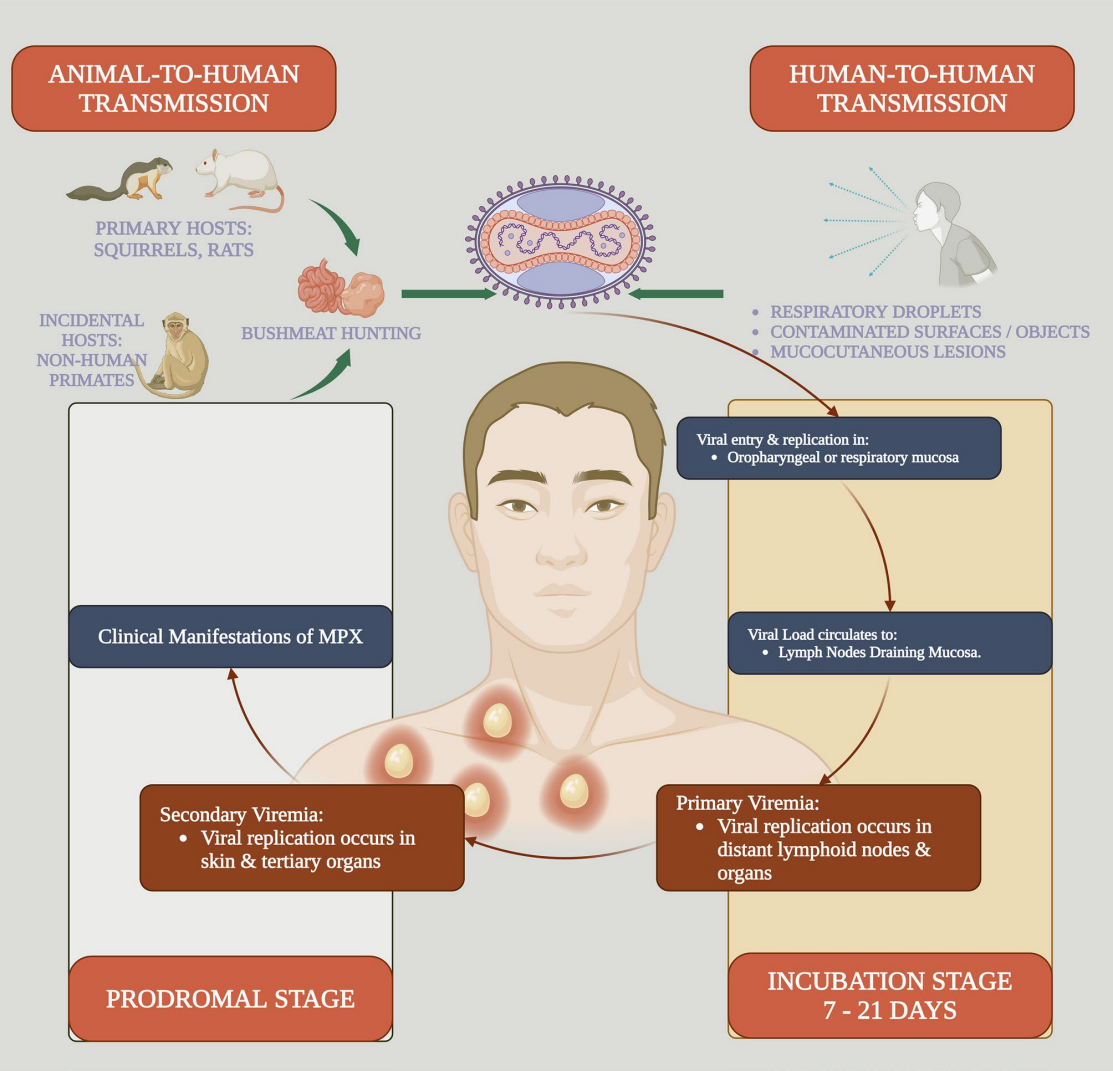
The pathophysiology of MPXV

Notably, the entry portal of MPXV is through subcutaneous injury and the naso-oropharynx. The MPXV multiplies at the site entry and then spreads to the regional lymph nodes [14]. Townsend, Keckler [15] showed that MPXV infection is associated with developing lymphadenopathy and lymphopenia. MPXV can evade immune detection by releasing pro-inflammatory cytokines that interfere with the expression of MHCI and CD4/CD8 receptors and intracellular signaling pathways [16]. Thus, MPXV can escape from immune detection in the early phase of infection. Furthermore, MPXV binds cell membrane glycosaminoglycan (GAG), which enhances viral endocytosis [17]. Viral proteins of MPXV are expressed within 48 h following infection in the Golgi apparatus and then transported through microtubules to the cell membrane again [18]. GAG is a complex carbohydrate expressed on the cell membrane and extracellular matrix that interacts with many pathogens, including MPXV [19]. MPXV activates the release of GAG, which coats the virus and prevents immune detection [20]. Hughes, Goldstein [21] found that heparin sulfate, sialic acid, and GAG, which are highly expressed in epidermal and dermal cells, facilitate the binding of MPXV. In addition, MPXV enters the host cell through the macropinocytosis process and multiplies in the cytoplasm only [22]. Multiplication of MPXV triggers the activation of helper T cells with weak T1h response, releasing pro-inflammatory cytokines such as IL-4, IL-5, and IL-6 [23]. These changes lead to primary and secondary viremia with the development of clinical presentations [Figure 2]. The clinical presentation of MPXV is characterized by fever, headache, myalgia, lymphadenopathy, and peripheral skin rash on the extremities that appears on the 3rd day of the disease. The mortality rate is low in mild infection; however, in severe illness caused by virulent strains, the mortality rate ranges from 10 to 17% [24, 25]. MPXV may cause severe complications, including bronchopneumonia, encephalitis, and vision loss, with mortality rates ranging from 10 to 17% in severe cases [26].

The first Outbreak of MPXV outside Africa was in 2003 due to contact with infected prairie dogs in the USA [27]. In 2017–2018, many outbreaks of MPXV in Nigeria led to 122 confirmed cases, with 6% mortality due to contact with infected rodents and domestic animals [28]. In 2022, the WHO declared a risk of MPXV epidemic in non-endemic countries such as Canada, Spain, and Portugal [29]. In 2023–2024, outbreaks of MPXV occurred in non-endemic areas, suggesting human-human transmission. In May 2024, more than 7000 cases were reported, more in children < 15 years, with 5.3% fatality [30]. In early August 2024, the MPXV outbreak rapidly extended in many African and non-African countries. Therefore, in August 2024, the WHO declared a global concern about the MPXV epidemic [31].

It has been shown that chronic viral infections such as coxsackievirus B, associated with the onset and the development of type 1 diabetes (T1D) by autoimmunity and direct β-cell death [32]. Comparable to other viral infections, MPXV may induce susceptibility to T1D [32, 33]. However, the molecular mechanisms for the induction of T1D in MPXV are not fully elucidated. Therefore, this review uniquely focuses on MPXV as a trigger of T1D, an area with limited exploration regarding the molecular mechanisms.

## Viral infections and T1D

**Fig. 3:**
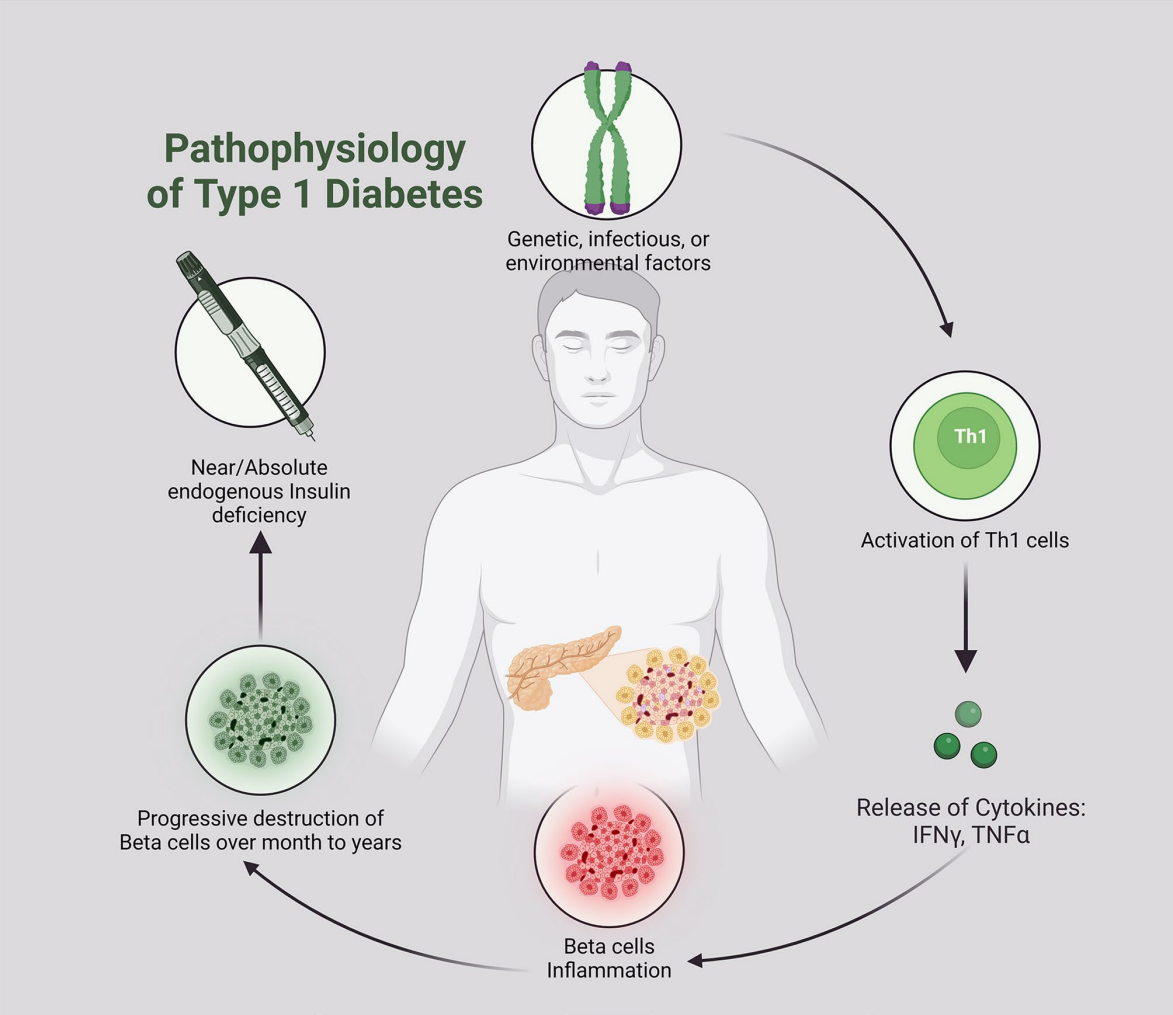
Pathogenesis of T1D

T1D, also known as autoimmune diabetes, is a chronic endocrine disease due to injury of pancreatic β cells leading to hyperglycemia [34]. T1D is the most common endocrine disease in children due to genetic and environmental factors [35]. In Western countries, the prevalence of T1D rises 3–5% per year [36]. T1D is clinically presented with hyperglycemia, polyuria, polydipsia, and weight loss. In addition, T1D patients may present with microvascular and macrovascular complications [35]. Pasi and Ravi [35] showed that 26.1% of T1D patients presented with diabetic ketoacidosis, and those presented with higher HbA1c had more complications. The pathogenesis of T1D is complex and related to different etiologies [Figure 3].

**Fig. 4:**
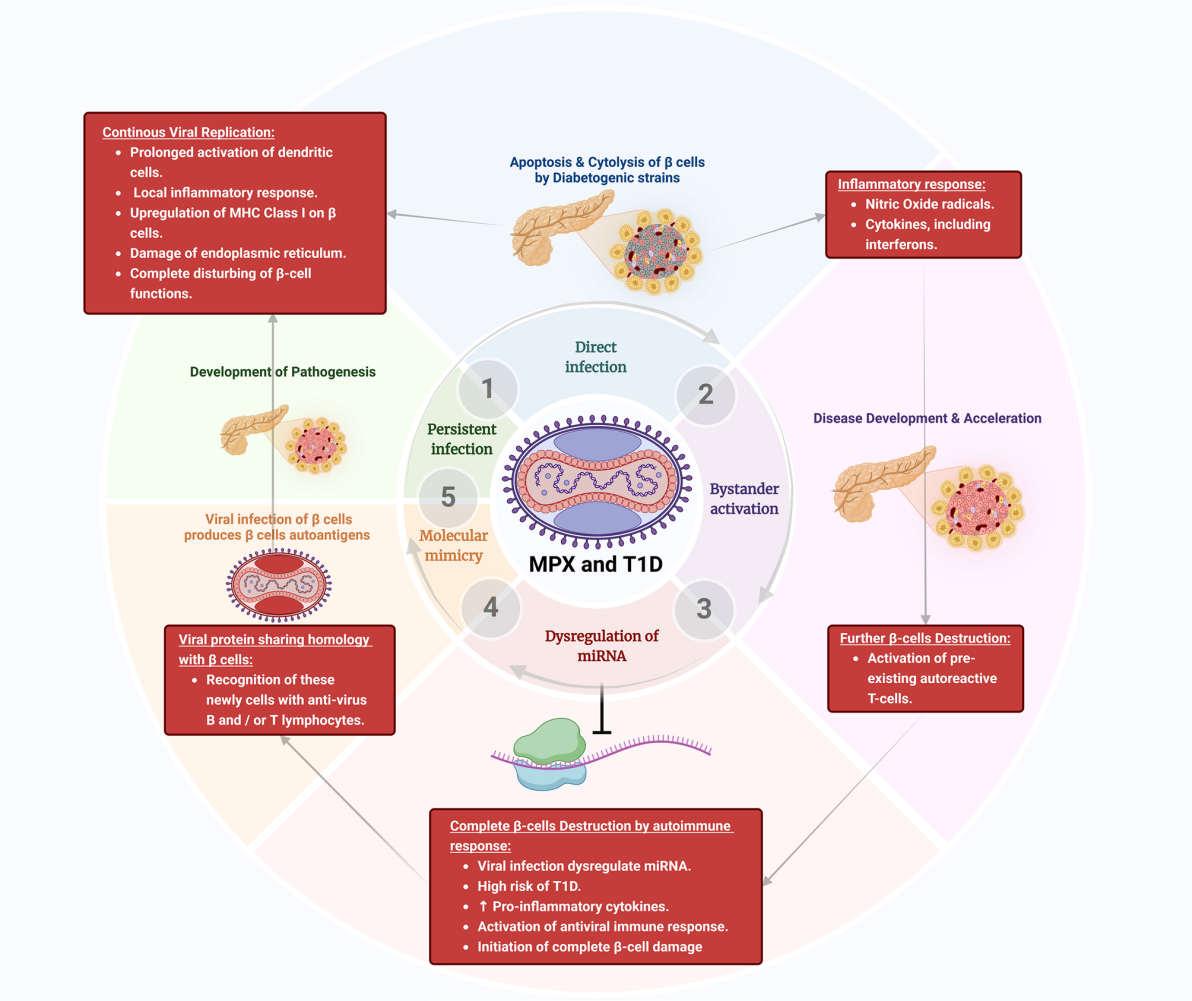
Viral infections and the development of T1D

The exact cause of T1D is not fully understood; however, T-cell damage to the pancreatic β cells is believed to be the primary mechanism [37]. Of note, the biomarkers of autoimmunity, such as islet-targeting autoantibodies, glutamic acid decarboxylase antibodies, and insulinoma-associated protein 2 are increased by months before the development of T1D symptoms [38]. The underlying causes of autoimmunity in T1D are multifactorial in genetically susceptible children [36]. Alteration of gut microbiota, dietary habits, and viral infections triggers autoimmunity in T1D. Houeiss, Luce [39] showed that viral infections augment T1D risk directly through injury of pancreatic β cells or indirectly by activating the immune system. Epidemiological studies confirmed that viral infections are associated with T1D development. Recent findings demonstrated that viruses, including MPXV, can bypass early immune detection during infection, leading to chronic inflammatory states that may exacerbate autoimmune conditions, such as T1D. This pattern was seen in viral infections such as cytomegalovirus (CMV) and Epstein-Barr virus (EBV), where prolonged immune activation led to significant β-cell destruction [40–42]. For example, enterovirus triggers autoimmunity and the injury of pancreatic β cells [40]. In addition, overexpression of the interferon (*IFN*) gene in the peripheral blood mononuclear cells (PBMCs) is augmented in susceptible children before the antibody response [41], suggesting activation of innate immune response before antibody response. Overexpression of *IFN*-stimulated genes is correlated with the development of insulitis [43]. Notably, many DNA viruses, such as CMV and EBV, and RNA viruses, such as enterovirus and rubella, are involved in the induction of T1D [44]. The duration between viral infection and the onset of an autoimmune response might vary from several months to several years, suggesting a prolonged impact of the viral infection [45]. It has been illustrated that recurrent viral respiratory infections by the age of 8 years are linked with an elevated risk of T1D [46]. This review draws on data from recent outbreaks of viral infections known to cause immune dysregulation, including COVID-19 and enterovirus, with MPXV due to an increase in its global presence. COVID-19 is associated with the development of T1D due to exaggerated immune response and destruction of pancreatic β cells [47]. These verdicts highlighted that viral infection through induction of autoimmunity provokes the development and progression of T1D [Figure 4].

Although enteroviruses and CMV are directly associated with T1D via β-cell cytotoxicity and autoimmune [40, 41], MPXV provokes T1D via sustained immunological dysregulation without acute cytopathic consequences. This indicates a specific mechanism by which MPXV may promote immunological tolerance and subsequent disruption, leading to the formation of autoreactive T cells. This route requires additional investigation to confirm its diabetogenic potential [16, 23].

## Core hypothesis: MPXV as a potential diabetogenic virus

### MPXV and pancreas

While direct clinical evidence linking MPXV to TID is limited, emerging research suggests a potential connection between MPXV and pancreatic dysfunction. In 2024, a recent study by Ritter, Martines [48] examined the development of necrotic lesions in the pancreatic tissues of patients with fatal MPX. In another recent case report, Paniagua-García, Casimiro-Soriguer [49] confirmed the presence of viral DNA in the pancreas. MPX is regarded as an opportunistic infection in patients with advanced HIV-associated immunosuppression. Interestingly, a preclinical study by Osorio, Iams [50] revealed that MPXV infection can lead to apoptosis of the pancreatic tissues in the BALB/c mice model. However, MPXV primarily affects lymphoid tissue, and tropism has also been reported in the pancreas. These findings proposed the negative impact of MPXV on the pancreas.

### MPXV and other diabetogenic viruses

MPXV, like other viral infections, increases the risk of developing T1D through binding cell membrane GAG [17], which is highly expressed in the pancreatic β cells [51], leading to direct inflammatory injury and the development of T1D. Recent data from COVID-19 indicate that viral-induced epigenetic alterations in pancreatic β-cells have a role in autoimmunity. Comparable effects may manifest in MPXV, necessitating more research to distinguish the diabetogenic effect of MPXV from other viral infections such as enterovirus and SARS-CoV-2 [33]. Many human and viral mimic epitopes, as in poxviruses, can mediate autoimmunity by inducing the development of autoreactive T cells through interaction with MHC [52].

Conversely, MPXV can release chemokine binding protein from infected cells that inhibits the release of macrophage inhibitory protein 1 (MIP-1), inhibiting chemokine functions and reducing the development of autoimmunity [53]. In addition, MPXV-infected cells prevent T-cell receptor-mediated T-cell activation [16]. Hence, the impact of MPXV on T cells is inhibitory in the initial stage of infection and stimulatory in the latter stage of disease. Overall, MPXV triggers abnormal immune tolerance with the development of late autoimmunity. Besides, uncontrolled inflammation and T cell-mediated autoimmunity are involved in the pathogenesis of T1D [54]. Macrophages and dendritic cells within the pancreas present antigens to autoreactive T cells, fueling an autoimmune response that damages pancreatic β cells. In response to inflammation caused by autoreactive T cells, the pancreatic β cells under stress increase the expression of MHC and co-stimulatory molecules, which may lead to further activation of autoreactive T cells [55]. Hence, a strong correlation exists between autoreactive T cells and pancreatic β cells at the onset of T1D progression. In an experimental viral-induced T1D, damaged pancreatic β cells by autoreactive T cells present more sequestered antigens with autoimmunity exaggeration [56]. Moreover, MPXV infection can cause the development of T1D by releasing pro-inflammatory cytokines and up-regulating inflammatory signaling pathways. Further studies are necessary to explore the potential impact of MPXV on glucose metabolism in diabetic patients.

### MPXV and Pro-inflammatory cytokines

**Fig. 5:**
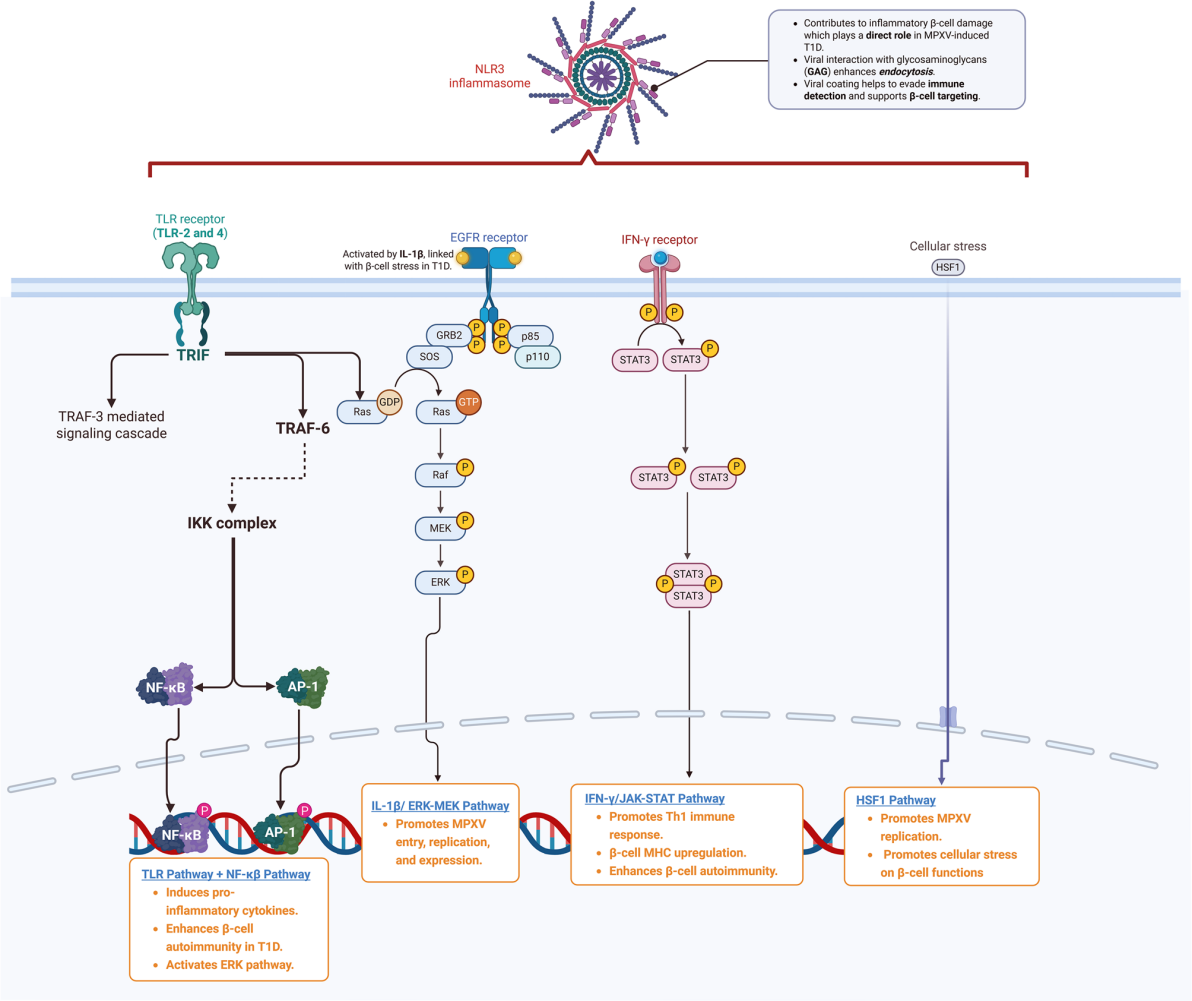
Key signaling pathways in MPXV infection

The inflammatory response induced by MPXV has been observed in preclinical and clinical settings, demonstrating cytokine release patterns like those seen in other autoimmune-triggering viral infections [Figure 5]. A recent comparative study on viral load and immune response in MPXV-infected macaques offers insights into how similar mechanisms may underlie MPXV-induced β-cell damage. Johnson, Dyall [57] showed that monocyte activation and the release of pro-inflammatory cytokines are augmented in early immune responses of MPXV. Preclinical findings demonstrated that MPXV mainly affects neutrophils and monocytes, though B cells and the natural killer (NK) cells are less affected [58]. In T1D, monocytes and neutrophils are modified in response to the effect of pro-inflammatory cytokines. Excessive activation of neutrophils and the release of pro-inflammatory cytokines reduce the protection against invading pathogens [59]. It has been stated that activated neutrophils and subsequent release of reactive oxygen species (ROS), pro-inflammatory cytokines, and formation of neutrophil extracellular traps (NETs) can initiate the diabetogenic process by injury of pancreatic β cells [60].

Furthermore, monocytes are involved in the pathogenesis of T1D. Monocytes isolated from T1D patients experience a spontaneous activation and release of pro-inflammatory cytokines compared to those isolated from healthy controls [61]. Pro-inflammatory cytokines from activated monocytes induce the activation of Th17 cells, which are implicated in the pathogenesis of T1D [62]. Therefore, uncontrolled activation of monocytes and neutrophils in MPXV may induce the pathogenesis of T1D. In addition, exaggerated immune response in MPXV [63] and the release of pro-inflammatory cytokines may cause injury of pancreatic β cells and the development of T1D [64]. A case-control study showed that the elevation of pro-inflammatory cytokines, oxidative stress, and T cell activation is correlated with the induction of the pathogenesis of T1D [65]. Suppressing the production of pro-inflammatory cytokines may reduce damage to pancreatic β cells [64]. Therefore, using anti-inflammatory agents in MPX may reduce the risk of developing T1D. Indeed, MPXV, via activation of NK cells, promotes the release of IFN-γ, which triggers an immune response by activating the Th1 immune response [15]. The overactive response of IFN-γ is associated with the severity of MPX [66]. Besides, dysregulation of the IFN-γ immune response is linked with the development of autoimmunity and progression of T1D [67]. It has been established that IFN-γ triggers abnormal immune signaling in the pancreatic β cells by inducing the activation of the JAK-STAT pathway and up-regulating MHC class I hallmark of autoimmunity and T1D [68, 69]. Also, IFN-γ promotes the homing and proliferation of autoreactive T cells [68]. Thus, dysregulation of IFN-γ due to abnormal immune response in MPX may induce the development of T1D.

MPXV induces distinct cytokine profiles during the first immune response, with IL-4, IL-5, and IL-6 primarily associated with MPXV infection [23]. This contrasts with the T1D cytokine profile in other viral infections, such as enterovirus, where IL-1β and TNF-α are more pronounced [40, 41, 70]. Such discrepancies may indicate a separate immunomodulatory role in MPXV-induced β-cell destruction, potentially influencing the development of T1D. Future experimental studies regarding β-cell-specific markers to track pancreatic damage following viral infection that provide further evidence of MPXV diabetogenic potential are recommended.

### MPX and inflammatory signaling pathways

It has been shown that inflammatory signaling pathways are activated in response to the MPXV infection, which triggers the development of inflammation by activating the release of pro-inflammatory cytokines. Of note, extracellular signal-regulated kinase (ERK) and mitogen-activated protein kinase (MAPK) are activated and involved in MPXV by increasing the entry, replication, and expression of MPXV proteins [71, 72]. In addition, the c-Jun N-terminal kinases (JNKs), as members of the MAPK family, which control cell proliferation and migration/invasion, are involved in COVID-19 and other viral infections, including MPXV [73]. It has been reported that IL-1β, which destroys pancreatic β cells, activates ERK and MAPK [70]. Activated ERK and MAPK are increased in macrophages of T1D patients [74]. ERK and MAPK are essential for insulin signaling in the pancreatic β cells; however, dysregulation of ERK and MAPK signaling is associated with injury of pancreatic β cells [75]. In addition, heat shock factor 1 (HSF-1) is implicated in the pathogenesis of MPXV by increasing the replication of MPXV. HSF1 is phosphorylated, translocated to the nucleus, and increases transcription of HSF1 target genes. The activation of HSF1 supported virus replication, as RNAi knockdown and HSF1 small molecule inhibition prevented orthopoxvirus infection. Consistent with its role as a transcriptional activator, inhibition of several HSF1 targets also blocked vaccinia virus replication. These data highlighted that orthopoxviruses co-opt host transcriptional responses for their benefit, extending their functional genome to include genes residing within the host DNA. The dependence on HSF1 and its chaperone network offers multiple opportunities for antiviral drug development [76]. Similarly, HSF-1, Toll-like receptors (TLRs), and heat shock proteins (HSPs) are the major metabolic pathways involved in the replication of MPXV in human and monkey cell lines [72]. These signaling pathways are also implicated in the pathogenesis of T1D. Dysregulation of heat shock stressors promotes immunological response and T cell activation during the pathogenesis of T1D [77]. At the onset of T1D, the cellular stress responsiveness is reduced due to an exaggerated inflammatory milieu [68].

Nevertheless, HSP90 is considered a sign of increased stress in pancreatic β cells, particularly in cases of T1D [78]. Therefore, dysregulation of HSPs in MPXV indicated pancreatic β-cell stress, which precedes the development of TID. Notably, TLRs, mainly TLR-2 and TLR-4, are up-regulated in the monocytes of T1D patients [79], signifying the involvement of these receptors in the pathogenesis of T1D. The interaction between TLRs and microbial ligands up-regulates the expression of pro-inflammatory cytokine mRNAs and triggers the destruction of pancreatic β cells. The up-regulated TLRs are synergized with viral infections to induce T1D development [80]. Therefore, exaggerated expression of TLRs in MPXV may cause the development of T1D.

Moreover, MPXV infection is associated with the expression and upregulation of nuclear factor kappa B (NF-κB) and nod-like receptor pyrin 3 (NLRP3) inflammasome [81, 82]. The NF-κB pathway is crucial for the functioning of autoreactive T cells and the development of T1D and other autoimmune illnesses. NF-κB promotes the release of pro-inflammatory cytokines from monocytes, resulting in the development of T1D [83]. The protein complex NLRP3 inflammasome is implicated in the pathogenesis of TID by inducing inflammatory destruction of pancreatic β cells [84]. It has been demonstrated that hyperglycemia can induce progressive injury of pancreatic β cells by activating the NLRP3 inflammasome by inhibiting AMP-activated protein kinase (AMPK) in INS-iE cells. Inhibition of NLRP3 inflammasome by vitamin D may attenuate the injury of pancreatic β cells. Supporting this claim, vitamin D improves pancreatic insulin signaling by inhibiting the NLRP3 inflammasome in diabetic patients compared to healthy controls [85]. In addition, sunlight exposure and vitamin D supplements reduce the severity of MPX and smallpox [86]. MPXV’s capacity to circumvent initial immune recognition by modulating MHC class I and CD4/CD8 receptors may distinctly predispose to persistent immunological responses, similar to other diabetogenic viruses [16, 17, 52]. This MPXV-specific mechanism necessitates more investigation into its diabetogenic potential.

Thus, MPXV could be a potential risk factor in the development of T1D by inducing an abnormal immune response and T cell-mediated autoimmunity. Therefore, early diagnosis and treatment of MPXV by anti-inflammatory and immunomodulator agents may decrease the risk of T1D.

## Comparative insights: lessons from other diabetogenic viruses

Although clear evidence connecting MPXV to T1D is still limited, the fast proliferation of MPXV and its similarity to the earlier viral pandemics need prompt inquiry. The insights gained from the COVID-19 pandemic, during which viral-induced autoimmunity markedly augmented T1D incidences [31, 87]. Therefore, prompt treatments with anti-inflammatory or immune-modulatory medications may mitigate the risk of T1D onset in persons infected with MPXV. Recent insights from COVID-19 treatment, particularly early cytokine regulation, may guide therapeutic strategies in MPXV patients with similar cytokine profiles and immunological responses [47, 67, 88]. By recognizing these connections, clinicians may implement preventive strategies for viral infections with autoimmune potential, mitigating public health concerns in future pandemics. Given the unexpected association between viral infections and autoimmune diseases in recent pandemics, MPXV could present new challenges in public health. Thus, continued research on the diabetogenic potential of MPXV is crucial for future pandemic preparedness.

## Conclusions

MPXV is a zoonotic disease endemic in Africa that can be transmitted to humans by direct contact with infected animals such as monkeys. MPXV infiltrates the host cell by macropinocytosis, replicates exclusively in the cytoplasm, and stimulates the activation of helper T cells, producing pro-inflammatory cytokines. These modifications result in the development of primary and secondary viremia, accompanied by clinical symptoms. Like other viral infections, MPXV may increase the risk of T1D. However, the fundamental mechanism for MPXV-induced T1D is not entirely elucidated.

MPXV activates abnormal immune tolerance with the development of late autoimmunity through the progression of autoreactive T cells. Uncontrolled inflammation and T cell-mediated autoimmunity have been regarded as the significant factors involved in the pathogenesis of T1D. MPXV infection triggers the development of T1D by inducing the progression of T cell autoimmunity, release of pro-inflammatory cytokines, and the upregulation of inflammatory signaling pathways. Moreover, MPXV infection is associated with the expression and upregulation of NF-κB and NLRP3 inflammasome, which are implicated in the pathogenesis of T1D.

While the evidence presented in this review suggests a potential link between MPXV and TID, further research is needed to establish a definitive causal relationship. Future studies should focus on gathering epidemiological data, conducting clinical trials, and investigating the underlying mechanisms by which MPXV infection may trigger autoimmune responses and pancreatic β-cell damage. This research will enhance our understanding of the long-term health consequences of MPXV infection and contribute to developing effective prevention and treatment strategies for MPXV-related complications, including T1D. Smallpox vaccines appear to be effective measures for managing MPXV outbreaks, though they do cause adverse effects, and second-generation replicative vaccines have prohibited usage. Third-generation vaccines pose a challenge for rapid responses as they require two doses, which can be difficult for people with fragile immunity [89].

Therefore, early diagnosis and treatment of MPX may decrease the risk of T1D. Utilizing anti-inflammatory and immunomodulatory medicines for different purposes might be a valuable strategy to reduce the occurrence of T1D in the next pandemic. Further studies are recommended in this regard.

## Data Availability

No datasets were generated or analysed during the current study.
